# PHYSICAL ASSESSMENT IN SURFERS: GUIDELINES FOR HEALTH PROFESSIONALS - PART 2 LOWER QUARTER

**DOI:** 10.1590/1413-785220253304e290579

**Published:** 2025-09-08

**Authors:** Guilherme Carlos Brech, Eduardo Takeuchi, Pedro Miguel Carriço de Seixas, Alexander Rehder, Marcus Vinicius Pereira Prada, Marcelo Baboghluian, Guilherme Vieira Lima

**Affiliations:** 1 Grupo de Estudos do SID (Surf Information Data), Sao Paulo, SP, Brazil.; 2 Personal Boards, Sao Paulo, SP, Brazil.; 3 Universidade de Sao Paulo (USP), Departamento de Ortopedia e Traumatologia, Laboratorio do Estudo do Movimento, Sao Paulo, SP, Brazil.; 4 Universidade Sao Judas Tadeu, Programa de Pos-Graduacao em Ciencias do Envelhecimento, Sao Paulo, SP, Brazil.; 5 Surfing Medicine International (SMI), Netherlands.; 6 Escola Superior de Saude Atlantica, Oeiras, Portugal.; 7 Faculdade de Medicina do ABC, Departamento de Ortopedia e Traumatologia, Cirurgia de Ombro e Cotovelo, Santo Andre, Sao Paulo, SP, Brazil.

**Keywords:** Practice Guideline, Water Sports, Musculoskeletal Pain, Knee, Hip, Ankle, Guia de Prática Clínica, Esportes Aquáticos, Dor Musculoesquelética, Joelho, Quadril, Tornozelo

## Abstract

Musculoskeletal injuries in the lower quarter during surfing are primarily associated with the sport's fundamental movements. This movements occur when the surfer is standing on the board, riding the wave. The injuries are typically acute, with severity and complexity varying according to the surfer's skill level and maneuvers performed. The objective of the present study was to conduct an integrative review of studies related to the musculoskeletal assessment of the lower quarter, focusing on physical examinations and functional tests applicable to surfers. This integrative review was carried out through a literature review, evaluating and analyzing papers from national and international journals indexed in the scientific databases Scielo and PubMed. The developement and analysis involved a panel of experts in the field of medicine and surfing health composed of physical educators, physiotherapists and sports doctors. This guideline aims to complement the information presented in the upper quarter article, emphasizing the prevalence of musculoskeletal injuries in the lower quarter among surfers and guiding outpatient assessments. It considers the specificities of surfing and the biomechanical movements involved. ***Level of Evidence III; Expert opinion.***

## RESUMO

As lesões musculoesqueléticas do quadrante inferior durante a prática esportiva do surfe, estão associadas principalmente ao gesto da modalidade esportiva. Gesto este relacionado ao momento em que o surfista se encontra em pé sobre a prancha, surfando a onda. As lesões apresentam características agudas, variando sua gravidade e complexidade conforme o nível de habilidade do surfista, em função das manobras realizadas. O objetivo do presente estudo foi realizar uma atualização referente aos estudos relacionados à avaliação musculoesquelética do quadrante inferior, relacionada ao exame físico e testes funcionais que possam ser aplicados ao surfista. Trata-se de estudo de atualização baseado em uma revisão integrativa através de levantamento bibliográfico no qual foram avaliados periódicos nacionais e internacionais indexados nas bases científicas Scielo e PubMed, desenvolvida e analisada por um grupo de especialistas na área de medicina e saúde do surfe composta por educador físico, fisioterapeutas e médicos do esporte. Este estudo de diretrizes buscou complementar as informações do artigo de quadrante superior, destacando a prevalência de lesões e avaliação musculoesqueléticas do quadrante inferior no surfista, norteando a avaliação ambulatorial do surfista, pensando na especificidade da modalidade e gesto biomecânico envolvido. ***Nível de Evidência III; Opinião do especialista.***

## INTRODUCTION

Surfing has become popular worldwide^1,2^ and even more in Brazil due to the recent achievements of professional surfers. With this growth, there is an increased exposure to the risk of injuries among practitioners.^3,4^

During surfing, the surfer needs physical skills such as balance, flexibility, muscle strength and agility.^2^ It is considered an intermittent activity, with the surfer spending approximately 50 to 60% of the time paddling, while lying on the board; 30 to 40% in a stationary phase, sitting on the board, and only 8 to 10% standing on the board performing maneuvers. The duration of this last phase can vary depending on the surfer's skill level ^5,6^

However, when the surfer is riding the wave, the musculoskeletal system of the lower limb, experiences great demand. At this stage of the activity, the prevalence of both acute and chronic injuries is directly related to the surfer's level of performance and skill.^7-12^

When analyzing the prevalence of musculoskeletal injuries in surfers, it is known that the lower limbs present a high incidence of involvement.^10,13,14^ All the demand placed on the lower limbs during surfing occurs within a closed kinetic chain.^15^ The structures of the lower limbs are subject to impact forces due to the explosive effort required for the movement and maneuvering the board. Additionally, the constant posture adopted during the sport, characterized by knee flexion and *valgus*, as well as the rotation of the femur relative to the fixed tibia imposes overload on static stabilizers of the knee, such as ligaments, joint capsule and meniscus. Consequently, an increase in the number of injuries has been observed among professional surfers in recent years.

Therefore, it is important to establish guidelines that can assist in the practice of evidence-based clinical evaluation for surfers in the outpatient setting. This includes assessments of the musculoskeletal system though physical examination and functional tests for the lower quarter, such as monitoring training load control, evaluating preventive strategies to minimize injury risk, and tracking the progression of treatment and performance.

## OBJECTIVE

The aim of this study is to provide an update on studies related to the evaluation of the musculoskeletal system in surfers’ lower quarter, focusing on physical examinations and functional tests.

## MATERIALS AND METHODS

This is an update study in which a bibliographic survey was conducted between June 2023 and July 2024 on Scielo and PubMed platforms, with terms related to surfing. However, as the scientific production on the theme is still quite scarce, the search has been expanded to support the construction of the physical evaluation guideline and functional tests in the surfer for the lower quarter.

### Clinical evaluation

Periodic clinical evaluations are routine for athletes and practitioners to enhance performance and prevent injuries, as they allow identification of opportunities for improvement and risk factors^16^. As noted in the upper quarter assessment, the goal is to determine the individual's physical and psychological health, providing a foundation for guidance and targeted interventions in training, nutrition, and mental health.

As surfers are exposed to highly variable environmental conditions, these assessments become indispensable. The clinical identification of conditions such as cardiomyopathies, valvopathies, hypertension, diabetes, hormonal imbalances, anemia, and others of any age are also essential^17,18^

### Hip

- **Epidemiology and Biomechanics**
  Scientific reports on hip injuries in surfers remain scarce. According to Hohn and colleagues^19^, hip injuries account for approximately 10% of all surfer injuries. In this retrospective study, it was observed that 62% of hip injuries require surgical intervention, with the average age of athletes undergoing surgery being 33.1 years. Femoroacetabular impingement (FAI) was identified as the most common injury to this joint, representing 67% of cases.
  A factor in the surfer's technique that may be associated with hip injuries could be related to the evolution of maneuvers. However, although an increase in hip injuries has been observed, this trend was not statistically significant, unlike the increase seen in ankle injuries.^19,20^
- **Structures at Risk**
  FAI is the most common injury mechanism, with the primary structures at risk being the femoral labrum and the acetabular rim, which may also involve cartilage defects. Additionally, injuries can occur in the bone marrow and round ligament, as well as joint impact, osteoarthritis, and loose bodies. Extra-articular structures that can also be affected by FAI related injuries include muscles and tendons, ischiofemoral impingement, trochanteric bursitis, iliopsoas tendinopathy, and strains of the hip adductors and flexors.^21^
- **Evaluation**
  The evaluation of surfers hip should be comprehensive, encompassing both extra-articular and intra-articular conditions. Hip mobility should be thoroughly assessed to identify potential restrictions that may predispose the athlete to injuries. Range of motion (ROM) tests are essential for determining joint flexibility and functionality.^20^
  FAI is a common condition among surfers, characterized by abnormal contact between the femoral neck and the acetabulum. There are three main types of FAI: CAM, PINCER, and COMBINED. The prevalence of FAI is estimated to affect around 10 to 15% of the population, making it a primary cause of hip pain in athletes.^21,22^ The clinical presentation varies, with young men being more prone to CAM type impingement and women more commonly affected by PINCER. The pain is typically located in the groin and sometimes lateral aspect of the hip, often exacerbated by repeated or prolonged bending, internal rotation, or abduction movements.^19,20^ Fadir and Faber tests may be positive, and anterior pelvic tilt may be present.^21,22^
  The strength and function of the gluteal muscles are essential for maintaining hip stability. Weakness in the gluteal medium and minimum can contribute to various painful conditions and hip dysfunctions.^22^

#### Extra-Articular Conditions

Pubalgia is a common condition in sports that involve repetitive, high-intensity movements, such as surfing. It is associated with hip muscle weakness (adductor strength less than 80%), abdominal weakness, reduced hip range of motion (ROM)^20^, and training specificity. During physical examination, assessments should include palpation, the squeeze test, ROM evaluation, and flexibility assessment.^21,22^

Ischiofemoral Impingement Syndrome (IFI) is an injury that affects the proximal tendons of the hamstrings and common in sports involving explosive and high-intensity movements.^21,22^ Assessing the strength and flexibility of the hip flexors is crucial for identifying muscle imbalances that may predispose individuals to injuries.^20^

#### Intra-Articular Conditions

These conditions may be associated with bone deformities that cause impact between the femur and the acetabulum, such as in FAI, or due to anomalies in hip development, like developmental dysplasia of the hip (DDH).^21,22^ In these cases, structural changes in the shape of the acetabulum can affect joint congruence^20^, potentially leading to micro instability either predisposed or acquired and damage to the round ligament.^21,22^ Additionally, acetabular labrum injuries can cause pain and instability and may be associated with cartilage damage and osteochondral injuries.^20^ Such cases may also present fragments of bone or cartilage within the joint, known as loose bodies.^21,22^

**III) Clinical Assessment**

The clinical evaluation of the surfer's hip should consider the demands, volume, and intensity of training.^21,22^ Addionally, it is important to assess genetic predisposition to certain conditions^20^ and anatomical variations that may increase the risk of injuries. Decreased range of motion of the hip, along with muscle weakness, can predispose individuals to a higher risk of injury.^21,22^

A thorough assessment and understanding of hip conditions is essential for the effective management of surfers’ injuries, enabling targeted and specific treatment strategies to enhance performance and prevent future injuries.

### Knee

**Figure 1:**
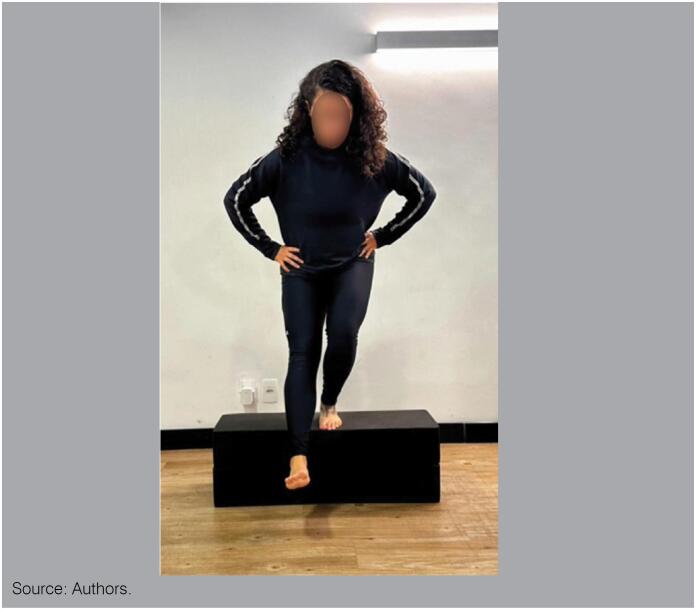
Step down test.

- **Epidemiology and Biomechanics**
  The knee is a joint that deserves special attention among surfers, as the incidence of injuries ranges from 10 to 28%.^9,23^ The increased incidence of these injuries has been observed in the last 15 years due to the change in pattern of maneuvers performed by athletes. Air maneuvers are the main responsible, especially at the time of landing, when the surfer's knee is subjected to a valgus stress force at the time of contact of the board with the feet, while landing on the unstable surface of the moving water.^19,24^
- **Structures at Risk**
  The main structures at risk are: medial collateral ligament (MCL), anterior cruciate ligament (ACL), and medial meniscus. Other less common are quadriceps and hamstring muscle injuries.^24,25^
  The study by Hohn and colleagues^19^ shows that in MCL injuries are more frequent for the lower limb positioned closer to the board's nose (63%), while for meniscal injuries the most affected limb is the one that is positioned closer to the board's tail (71% of cases).
- **Clinical evaluation**
  Musculoskeletal evaluation of the knee should address flexion and extension mobility for both knees. Knee extension deficits may be related to muscle shortenings, which can directly impact performance and increase the risk of injury in athletes.^26^
  The static physical examination should be carried out bilaterally, initially evaluating the stability of the knee, taking into account the ligament and meniscal structure. *Valgus* and *varus* stress test to evaluate side ligaments. Previous drawer tests, Lachman or Pivot Shift, to evaluate the ACL. For meniscus evaluation, McMurray and Apley tests can be used.^27^
  The dynamic evaluation of the knee should take into account the dynamic knee valgus (internal femur rotation + knee valgus), which may present weakness of hip stabilizers, and may be associated with the increased risk of knee injuries, especially the ligaments. This fact may also be aggravated by progressive maneuvers performance (e.g. aerials), which require some degree of impact and instability while landing, as well as repeated back-foot movements, with sharp valgus during the critical maneuvers. It si known that to improve the motor behavior of dynamic knee valgus, exercise programs aimed at greater muscle activation of the hip muscles are necessary,^28,29^ which can facilitate strategies capable of minimizing injury risks or correction of the sporting gesture.^30^
  For the clinical diagnosis of dynamic knee valgus, we can use the *Step Down* test that qualitatively evaluates the closed-chain kinetic stability of hip stabilizers. It only needs a 20 cm step. It should be observed the presence of contralateral pelvic fall or elevation, ipsilateral hip induction or internal rotation and ipsilateral knee valgus. This is a qualitative assessment that qualifies the movement in: high risk (when the patella moves inward, ending medially at the first finger of the foot); and low risk (when the patella ends in line with the first finger of the foot or laterally).^31,32^ You can also use a camera to record and assist movement analysis to compare the movement. (Figure 1)

### Ankle and foot

- **Epidemiology and Biomechanics**
  Ankle injuries are very common in general sports practice. In surfing, the incidence of injuries in this joint compared to practices such as basketball, volleyball and football is much lower. According to Furness and colleagues,^33^ the incidence of acute ankle injuries was 14.6% in 1348 surfers who participated in the study, with the main mechanism being direct trauma in 54.6% of cases, followed by stumbling's caused by base maneuvers in 30.6% of cases, and by aerial maneuvers in 13.9% of cases. In this study, the authors did not demonstrate a difference between professional and recreational surfers. Taylor and colleuagues, in their study with 668 surfers, demonstrated an incidence of 17.8% ankle and foot injuries, comprised by lacerations (n=17); sprains (n=10) and fractures (n=3).^14^ As for the most affected ankle in the surfer, the study of Hohn nd colleagues^19^ observed that the back-foot ankle is committed in 73% of the incidents. Another problem is the repetitive wrinkle, where up to 80% of athletes will have recurrent wrinkles and up to 72% may develop chronic ankle instability.^34^
  In recent years, we have observed a greater incidence of ankle injuries in surfers who are able to perform the aerial maneuver. Several authors point out the importance of the execution of this maneuver for the significant increase in the scores in competitive surf, however it is noteworthy that the success rate of aerial maneuvers in competitions is below 50%, and consequently, the risk of injury is also elevated.^35^ Even if the surfer is unable to perform the aerial, surfing a wave requires continuous and relatively rapid force production and stopping, especially in the lower body.^36^
  In addition to the sprain, direct trauma of the foot with the board or with the seabed, can cause fractures, being metatarsal fractures the most frequent in the practice of surfing.
- **Structures at risk**
  The main structures at risk in ankle injuries in surfers are the bone and stabilizing structures of the ankle and foot. The surfer may have Lisfranc joint luxation and fractures and metatarsal fractures, which are often neglected. Injuries in the ankle joint can compromise the subtalar joint, as well as more complex ligamentar ankle injuries such as syndesmosis.^24,37^
- **Clinical Assessment**
  The American Orthopedic Foot and Ankle Society presents an instrument called the Ankle-Hindfoot Scale (AOFAS) which is a clinical evaluation tool used primarily by health professionals to evaluate the function, pain and alignment of the foot and ankle, and is often used to evaluate the effectiveness of treatments and interventions for foot and ankle conditions, the scale has been translated and adapted with reproducibility and reliability for Portuguese.^38^
  In the physical examination, physical tests should be conducted to evaluate the amplitude of movement and muscle strength, joint stability and function of the muscles and ligaments of the ankle and foot.^39^ In cases of ankle sprain or instability it becomes important to evaluate the lateral ligamentary complex (mainly the anterior talofibular ligament and calcaneofibular). In cases of history of sprains with greater energy and complexity need to investigate the ankle joint syndesmosis. Balance tests are also important to evaluate the patient's ability to maintain balance and awareness of ankle and foot position.^40^

#### Functional tests and computerized analysis

Functional tests are mainly used for functional evaluation of the knees and ankles. Functional performance tests are useful predictors of lower extremity performance.

Localized ankle instability and chronic sprains can directly impact postural control, functional deficiencies have been identified, which can generate imbalances not only in the ankle,^34,41,42^ as well as the inverse is also used (general stability) for the development of proprioceptive control, neuromuscular and balance training, which considerably reduce the risk of recurring sprains in the ankle.^43-45^ During the aerial maneuver, inefficiency in the absorption of the forces generated at landing can result in acute injuries in the lower limbs.^36^

A similar mechanism occurs in improper basketball landings, directly linked to patellar tendinopathy.^46^

In landing, the joints of the lower end work together to relieve the impact forces. A lower amplitude of ankle dorsiflexion movement in the landing is associated with a lower knee flexion and greater ground reaction loads, enabling greater valgus knee displacement and increasing the likelihood of injury.^47^ According to Lundgren et al.,^48^ ankle dorsiflexion movement amplitude can be an important factor in allowing the surfing athlete to perform an aerial landing with movement amplitude, decreasing peak force and reducing direct joint load and consequently decreasing the likelihood of injury.

#### Lower Quarter Y-Balance Test

The Lower Quarter Y-Balance Test (YBT), also known as the Y-Test, is a dynamic balance test that requires strength, flexibility and proprioception. It aims to evaluate the risk of injury to the lower limbs and analyze the evolution of treatment.^49^ It is a test that allows to evaluate the ankle, knee and hip, evaluating the dynamic stabilization in the three directions: anterior, posteromedial and posterolateral.^50^ The goal of the test is to maintain the position on a single leg and exercise the maximum extension force of the contralateral leg seeking the greatest range while keeping the dynamic postural balance.^49^ During the execution of the test the surfer should push the target over the tube as far away as possible, keeping the hands on the hip and the test calcaneum standing on the platform. Distance is computed. The values are discarded if the surfer does not maintain the posture on the platform, or loses foot contact with the target during the movement of the same or if the foot has not returned to the initial posture.^49,51^ It is recommended that you make nine attempts (three for each direction), being only the first six for familiarization. For data normalization should be used the calculation of the *distance* obtained in an attempt x 100/ relative length of the limb.

**Figure 2:**
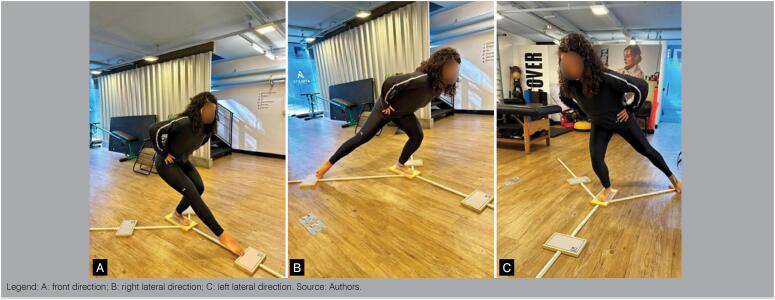
Lower quarter y-balance test.

To evaluate the performance of the surfer in the test should be considered the sum of the three reach directions, and the score is calculated from the total distance of the excursion dividing by three times the length of the limb (Figure 2). The length of the limb consists of the distance measured from the anterior iliac spine to the top of the external malleolus.^52^ In this sense, the test parameters can be both comparative (for treatment progression) and potentially predictive of risk of injury, if interlimb difference on the anterior reach distance is < 94%.

#### Hop e Single-Legged Hop Tests

The battery of tests called Single-Legged Hop Tests, developed by Fitzgerald et al.^53^ has been widely used in clinical practice as a tool to evaluate neuromuscular control. These tests involve directional shifts and acceleration-deterioration movements, replicating the sporting demands specific to the knee and ankle of surfers.^54,55^ Assess the jumps with the greatest range possible, keeping balance and stability during landing.

The Single-Legged Hop Tests comprise four types of jumps: single-leg hop, triple hop, cross-over hop, and 6-meter timed hop.^56^ These tests are focused on evaluating the individual's muscle strength, stability, and ability to maintain posture during repeated and rapid side movements.^55^

The Side Hop Test aims to evaluate agility, coordination and ability to maintain ankle control and stability during movements that require rapid change of direction. Participants jump laterally, on a 5-meter course delineated by cones, forming an eight-shaped pattern. The Crossover Hop Test is designed to evaluate the individual's ability to perform fast and controlled diagonal movements, challenging lateral stability and ankle coordination. Participants jump diagonally over a 6 meters long and 15cm wide line, alternating sides along the line as quickly as possible. The Square Hop Test evaluates the agility and ability to perform fast and precise movements in various directions, in a square of 40×40 cm, for 5 repetitions as quickly as possible. The results of these tests can be expressed in two ways: the distance reached or the time spent in the case of the *6-meter timed hop*, or through an index of symmetry of the lower limbs (ISMI).^55^ The ISMI reference value, which indicates significant differences in symmetry between the lower limbs, is 90%. Larger differences may indicate a deficit in neuromuscular control and early detection of possible imbalances that can lead to injuries and affect athletic performance.^55^

### Weight-Bearing Lunge Test

The Weight-Bearing Lunge Test (WBLT) is a clinical test that evaluates the ROM of ankle joint dorsiflexion in closed kinetic chain. It is used to detect movement amplitude deficits in people with chronic ankle instability and monitor progress in improving movement amplitude during rehabilitation protocols.^58,59^

**Figure 3:**
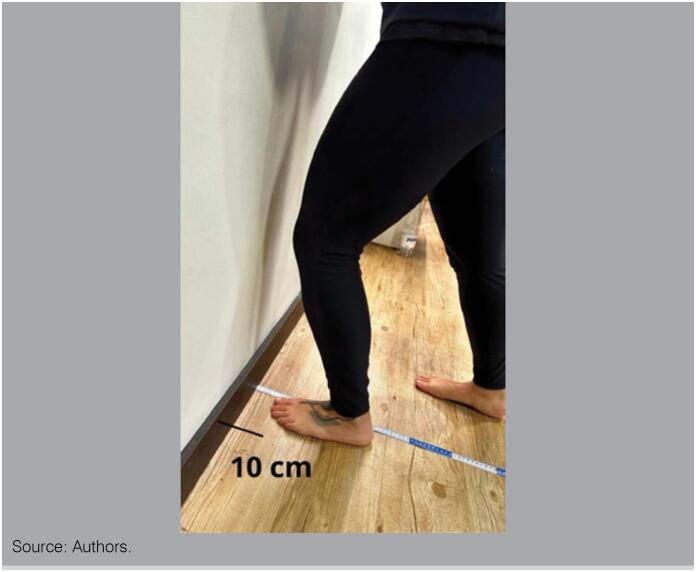
Weight-bearing lunge test.

WBLT is performed standing, with the heel in contact with the 56th floor, the knee aligned with the second finger of the foot and the toe 10 cm away from the wall. (Figure 3)

The participant proceeds trying to touch a vertical line on the wall with his knee, keeping the heel in contact with the ground. If you can lean your knee against the wall while keeping both the heel and knee in contact with it, then move the foot 1 cm away from the wall and lean forward until reaching the maximum dorsiflexion range, the maximum ankle dorsiflexion angle can be evaluated.^59,60^

#### Computerized analysis

The computerized evaluation in the surfer is more relevant for professional surfers who need to improve performance, or as a thorough investigation of possible imbalances that may increase the risk of injury in the sport. In addition, it is an excellent resource for determining the return to sport after a rehabilitation process that involves the restoration of muscle capacity.^61^ In this sense, we can highlight two valuable instruments for the evaluation of skeletal muscle systems. The evaluation in the isokinetic dynamometer of strength, potency and muscle endurance is excellent for quantifying possible discrepancies between muscle groups.^62^ In this sense, it becomes important to conduct an annual evaluation of the professional surfer. Another important feature for evaluating the muscle strength of the limbs is the counter-movement jump, using a contact plate or force platform. The importance of evaluating the dynamic strength of the lower body is that stronger surfers appear to be able to develop a significantly greater eccentric peak speed,^63^ brake more efficiently and better use the landing/excentric stage, optimizing their jump performance.^64^

It is important to highlight that the risk and type of structures involved in the lesions of the lower quarter in the surf, are mainly related to the surfer's performance level. For often the acute lesions of the lower quarter of ligamentary or bone origin, are related to more complex maneuvers, such as the aeria" that are performed by more experienced and radical surfers.^24^ The bone injuries caused by direct trauma with the sea floor, are often associated with less experienced surfers.

The present study presents limitations because it is a review study with a scarce literature so far on the specific topic related to the sport. In addition, sport is undergoing transformations in recent years/decades regarding changes in the style of surfing, and as to the maneuvers performed. These changes have required greater overload of the structures of the lower limbs both amateur and professional surfers. This fact is impacting on an increase in musculoskeletal lesions in these segments. Moreover, the development and evolution of surfing in a more controlled environment (i.e. wave pools) can also present changes in the profile of injuries of practitioners who have access to this type of environment, which have to be considered and subject of future studies.

## FINAL CONSIDERATIONS

Surfing lacks of scientific evidence, mainly related to the orientation of surfer health and physical assessment. These assessements might be important, by offering new scopes for valid and reliable measurements, treatment progression options and injury prevention strategies . Therefore, the present study aimed to bring a direction for evidence-based clinical practice, providing guidance for the healthcare professional in ambulatory setting, for the musculoskeletal lower quarter assessment, aiming at the specificity of the level and characteristic of the surfer.
